# Effects of Reducing Suppressors of Cytokine Signaling-3 (SOCS3) Expression on Dendritic Outgrowth and Demyelination after Spinal Cord Injury

**DOI:** 10.1371/journal.pone.0138301

**Published:** 2015-09-18

**Authors:** Keun Woo Park, Ching-Yi Lin, Kevin Li, Yu-Shang Lee

**Affiliations:** Department of Neurosciences, Lerner Research Institute, Cleveland Clinic, Cleveland, Ohio, United States of America; University of Louisville, UNITED STATES

## Abstract

Suppressors of cytokine signaling-3 (SOCS3) is associated with limitations of nerve growth capacity after injury to the central nervous system. Although genetic manipulations of SOCS3 can enhance axonal regeneration after optic injury, the role of SOCS3 in dendritic outgrowth after spinal cord injury (SCI) is still unclear. The present study investigated the endogenous expression of SOCS3 and its role in regulating neurite outgrowth *in vitro*. Interleukin-6 (IL-6) induces SOCS3 expression at the mRNA and protein levels in neuroscreen-1 (NS-1) cells. In parallel to SOCS3 expression, IL-6 induced tyrosine phosphorylation of signal transducer and activator of transcription 3 (STAT3) in NS-1 cells. Lentiviral delivery of short hairpin RNA (shSOCS3) (Lenti-shSOCS3) to decrease SOCS3 expression into NS-1 cells enhanced IL-6-induced tyrosine phosphorylation of STAT3 (P-STAT3 Tyr705) and promoted neurite outgrowth. In addition, we determined if reduction of SOCS3 expression by microinjection of Lenti-shSOCS3 into spinal cord enhances dendrite outgrowth in spinal cord neurons after SCI. Knocking down of SOCS3 in spinal cord neurons with Lenti-shSOCS3 increased complete SCI-induced P-STAT3 Tyr705. Immunohistochemical analysis showed that complete SCI induced a significant reduction of microtubule association protein 2-positive (MAP-2+) dendrites in the gray and white matter at 1 and 4 weeks after injury. The SCI-induced reduction of MAP-2+ dendrites was inhibited by infection with Lenti-shSOCS3 in areas both rostral and caudal to the lesion at 1 and 4 weeks after complete SCI. Furthermore, shSOCS3 treatment enhanced up-regulation of growth associated protein-43 (GAP-43) expression, which co-localized with MAP-2+ dendrites in white matter and with MAP-2+ cell bodies in gray matter, indicating Lenti-shSOCS3 may induce dendritic regeneration after SCI. Moreover, we demonstrated that Lenti-shSOCS3 decreased SCI-induced demyelination in white matter of spinal cord both rostral and caudal to the injury site 1 week post-injury, but not rostral to the injury at 4 weeks post-injury. Importantly, similar effects as Lenti-shSOCS3 on increasing MAP-2+ intensity and dendrite length, and preventing demyelination were observed when a second shSOCS3 (Lenti-shSOCS3 #2) was applied to rule out the possibilities of off target effects of shRNA. Collectively, these results suggest that knocking down of SOCS3 enhances dendritic regeneration and prevents demyelination after SCI.

## Introduction

Spinal cord injury (SCI) encompasses primary mechanical damage and subsequent secondary degenerative responses [1–3]. Primary mechanical damage induces a cascade of excitotoxicity, oxidative stress, and membrane breakdown that, in turn, triggers neuronal cell death, axonal/ dendritic loss, and secondary degeneration [4–7]. Secondary degenerative responses include axonal loss and oligodendrocyte death, which may contribute to demyelination of spared axons after SCI [8]. In order to repair injured spinal cord, nerve regeneration strategies have been developed to overcome the inhibitory environment or to enhance nerve growth capacity after SCI. Most of these studies have focused on how to promote the regeneration of axons in both descending and ascending projections after SCI [9, 10]. However, the distribution of dendrites from local spinal cord neurons and how to promote the growth of these dendrites after SCI are still unclear. The extension of dendrites in the white matter from spinal cord neurons receives inputs from descending pathways [11]. The distribution of dendritic trees and the location of soma in certain spinal cord neurons may correlate with their function [12, 13]. Therefore, in addition to long tract regeneration, the growth of dendrites from local spinal cord neurons and the maintenance of dendritic distribution in the white matter may play important roles in repairing the injured spinal cord.

Suppressors of cytokine signaling (SOCS) proteins function in a negative feedback loop to terminate signaling through the Janus kinase (JAK)/ signal transducer and activator of transcription (STAT) pathway [14, 15], which regulates neuronal growth and differentiation [16, 17]. Suppressors of cytokine signaling-3 (SOCS3), one member of the SOCS family of proteins, binds to gp130, a common receptor for signal transduction with interleukin-6 (IL-6), or to JAK1 and JAK2, subsequently inhibiting signal transduction [18, 19]. Expression of SOCS3 in neurons plays a negative role in regulating cell survival and neurite outgrowth [20–23]. These studies, including our recent study [20], have demonstrated that cytokine- or nerve injury-induced SOCS3 expression negatively regulates the activity of STAT3, consequently leading to reductions in neuroprotection and neurite outgrowth. However, the mechanisms underlying the regulatory effects of SOCS3 on dendritic growth after complete SCI are still not clear.

In the current study, we hypothesized that SOCS3 expression in neurons negatively regulates neurite outgrowth *in vitro* and dendrite outgrowth after SCI. We determined the expression pattern of SOCS3 and its regulatory effects on neuritic outgrowth through STAT3 signaling *in vitro* by using neuroscreen-1 (NS-1) cells. We also investigated whether SOCS3 expression regulates dendritic arborization and demyelination after complete SCI in adult rats. Our results show that SOCS3 expression, in response to IL-6 treatment, negatively regulates neurite outgrowth *in vitro* via STAT3 signaling and that a reduction of SOCS3 expression by Lenti-shSOCS3 in spinal cord neurons enhances dendritic regeneration in the white matter and prevents demyelination after complete SCI.

## Materials and Methods

### Cell Cultures and Lentiviral Infection

NS-1 cells (Thermo Fisher Scientific, Pittsburgh, PA, USA), a PC12 subclone, were grown in RPMI medium containing 10% horse serum, 5% heat-inactivated fetal bovine serum, 1% L-glutamine, and 1% penicillin/streptomycin (EMD Milipore Co., Hayward, CA, USA). Cells were plated on 12-mm round glass coverslips or 6-well plates pre-coated with collagen I (10 μg/ml, Sigma-Aldrich, St. Louis, MO, USA) at a density of 1.0 × 10^3^ cells/coverslip or 5.0 × 10^4^ cells/well, respectively. NS-1 cells were incubated with NGF (2 ng/ml, R&D Systems, Minneapolis, MN, USA) in the culture media for 48–72 h after plating and then infected with lentivirus containing pGipz (Lenti-pGipz) or shSOCS3/pGipz (Lenti-shSOCS3) at a multiplicity of infection (M.O.I) of 40. After infection, puromycin was added for 2 days to select the lentivirus-infected NS-1 cells. Cultured cells were treated with IL-6 (100 ng/ml) for the designated time periods and then harvested for analyses of RNA and protein expression.

### RNA Isolation and Quantitative Real Time-PCR

Cultures treated with IL-6 were washed with RNase-free PBS and total RNA was extracted using Trizol (Invitrogen, San Diego, CA, USA). Purified RNA (500 ng) was reverse transcribed into cDNA using Multiscribe reverse transcriptase and random primers (Applied Biosystems by Life Technologies, Grand Island, NY, USA). Quantitative real-time PCR (qPCR) was performed as previously described to determine levels of SOCS3 mRNA [20, 21]. Data were analyzed using the comparative cycle threshold method to obtain quantitative values.

### Immunoblotting of NS-1 Cells

IL-6-treated cultures were lysed in buffer containing the following: 150 mM NaCl, 10 mM Na_2_HPO_4_ (pH 7.2), 0.5% sodium deoxycholate, 1% NP-40, and protease inhibitor mixture. Forty μg of total cell lysate were separated by electrophoresis on 8% SDS-polyacrylamide gels and blotted with the following antibodies: SOCS3 (1:2,000; abcam, Cambridge, MA, USA), tyrosine phosphorylation of STAT3 (P-STAT3 Tyr705) (Cell Signaling Technology, Danvers, MA, USA), β-actin (1:4,000; Cell Signaling Technology), or STAT3 (1:1,000; Cell Signaling Technology) as described previously [20]. Immunoreactivity was assessed using Pierce ECL or SuperSignal West Dura substrate (Thermo Scientific, Rockford, IL, USA). For quantitative analyses, band densities on immunoblots were measured with ImageJ software [20].

### Measurement of Neurite Length in NS-1 Cells

NS-1 cells were fixed with 4% paraformaldehyde after treatment, permeabilized with 0.2% Triton X-100 for 15 min, washed with PBS, and then stained with HCS CellMask Red (0.5ug/ml, Invitrogen) for 30 min at room temperature. Images were taken using a fluorescent microscope (DM6000; Leica Microsystems, Buffalo Grove, IL, USA). For measurement of neurite length, images were randomly selected and taken using a fluorescent microscope (DM6000; Leica Microsystems). The length of neurites of 150–200 single cells per condition was analyzed with LAS AF software (Leica Microsystems).

### Animal Groups

Adult female Sprague-Dawley rats (220–250g; Harlan Laboratories, Madison, WI, USA) were assigned randomly into three groups: (1) sham control (laminectomy only; con group; total n = 12); (2) T8 spinal cord transection (Tx) with Lenti-pGipz injection (Tx + pGipz group; total n = 14); or (3) T8 spinal cord transection and Lenti-shSOCS3 injection (Tx + shSOCS3 group; total n = 19). Rats were housed in standard laboratory cages under 12:12-hour light-dark cycle conditions with standard rodent chow and water available *ad libitum*. All experiments were performed during the light cycle. All animal procedures were approved by the Cleveland Clinic Institutional Animal Care and Use Committee (IACUC; protocol number: 2013–0973). The surgery was performed under isoflurane anesthesia with all efforts made during post-operative care to minimize suffering. All procedures were performed in strict accordance with the recommendations in the Guide for the Care and Use of Laboratory Animals of the National Institutes of Health.

### Lentiviral Production and Delivery to the Spinal Cord

Two lentiviral plasmids provided by Dr. Etty Benveniste (University of Alabama at Birmingham), shSOCS3/pGipz, and shSOCS3 #2/pGipz, encoding two shRNAs specific for SOCS3 to knockdown SOCS3 expression, were used to clarify the effects obtained were specifically mediated by reduced SOCS3 expression, rather than off target effects of shRNA [24]. Lentiviral particles were generated by calcium phosphate-mediated co-transfection of HEK-293T cells with shSOCS3/pGipz, shSOCS3 #2/pGipz, or empty pGipz together with psPAX2 (Packaging plasmid) and pMD2G (Envelope plasmid). Lenti-shSOCS3, Lenti-shSOCS3 #2, or Lenti-pGipz was collected after 72 h with titers up to 3–4×10^9^ infectious units/ml as previously described [20, 21]. Lentiviral delivery into the spinal cord was performed 2 weeks before T8 spinal cord transection or sham procedures. Animals were anesthetized by 2% isoflurane gas mixed with oxygen and then a laminectomy was performed at the T8 level, followed by insertion of a pored glass pipette attached to a microinjector into the gray matter of the spinal cord. The target areas for injection included four total sites with 2 mm depth at the following coordinates: two sites 2.5 mm rostral and two sites 2.5 mm caudal from injury site. Infusions were made at a rate of 133nl/min for Lenti-pGipz, Lenti-shSOCS3, or Lenti-shSOCS3 #2 (2×10^7^ total infectious units in 4 μl). After injection, the glass pipette was left in place for an additional 2 min before being slowly retracted.

### T8 Complete Spinal Cord Transection in Lentivirus-Infected Animals

All surgical procedures were conducted under aseptic conditions two weeks after lentiviral infection [20]. All lentivirus-infected animals were anesthetized with 2% isoflurane gas mixed with oxygen and were placed on a heating pad to maintain body temperature within ± 1.5°C of 36.5°C during surgery. In the Tx group, a laminectomy was performed, followed by complete transverse cuts of the spinal cord at the T8 level, resulting in a gap of 1~2 mm. A surgical microscope was used to ensure no neural tissue remaining in the gap. The bladders of all spinal cord-transected rats were expressed manually twice per day throughout the experimental period. The observation period was 1 or 4 weeks after complete SCI.

### Immunoblotting of Spinal Cord Tissue

At the designated time point after SCI, spinal cord tissues were obtained from areas 4 mm rostral or caudal to the injury epicenter and were homogenized in ice-cold lysis buffer as described previously [20]. Equal amounts of protein (40 μg) were separated by SDS-PAGE and blotted with antibody against GAP-43 (1:1000; abcam), β-actin (1:4,000; Cell Signaling Technology), or microtubule association protein-2 (MAP-2) (1:2000; Sigma-Aldrich). Immunoreactivity was assessed using Pierce ECL or SuperSignal West Dura substrate (Thermo Scientific). For quantitative analyses, band densities on immunoblots were measured with ImageJ software [20].

### Immunostaining of Spinal Cord Tissue

In parallel experiments, animals were anesthetized at 1 or 4 weeks after SCI or sham procedures and then transcardially perfused with 0.9% saline followed by 4% paraformaldehyde. Spinal cord were removed, post-fixed overnight at 4°C with 4% paraformaldehyde, and then incubated at 4°C with 30% sucrose solution until sinking. The spinal cord segments 4 mm rostral or caudal to the injured site were then transversely sectioned on a cryostat (30 μm) and collected for immunohistochemistry [25]. Briefly, free-floating serial sections were washed three times for 10 min with PBS and blocked with PBS containing 3% normal horse serum and 0.25% Triton X-100 for 1 h at room temperature. After washing with PBS, sections were then incubated overnight with gentle agitation at room temperature with antibodies against GAP-43 (abcam) and/or MAP-2 (Sigma-Aldrich). Sections were then washed with PBS and incubated for 1 h at room temperature with a secondary antibody conjugated by Alexa Flour 488 or 594 as appropriate (1:2,000; Life Technologies). Tissues were then washed and mounted with Vectashield mounting medium (Vector Laboratory, Burlingame, CA, USA). Sections were examined and all images were taken using a fluorescent microscope (DM6000; Leica Microsystems).

### Measurements of MAP-2 Positive (MAP-2+) Dendritic Intensity in Spinal Cord

The intensity of MAP-2+ immunoreactivity in spinal cord was assessed by examining sections obtained from areas 4 mm rostral or caudal to the injury site. One of every six serial sections (180 μm apart) were selected and immunostained with MAP-2 antibody (Sigma-Aldrich). Digital photomicrographs of MAP-2+ images were then taken with a Leica microscope (DM6000; Leica Microsystems) and analyzed with LAS AF software (Leica Microsystems) to measure MAP-2+ immunoreactivities in both gray and white matter of spinal cord. A total of 15–18 sections in each area rostral and caudal to the injury site were measured and analyzed.

### Measurements of MAP-2+ Dendritic Length in the White Matter of Spinal Cord

As described above, the lengths of MAP-2+ dendrites in both ventral horn and dorsal horn were measured after immunostaining of spinal cord tissues with MAP-2 antibody. The MAP-2+ dendrites were manually traced with a Wacom tablet only from the edge of gray matter and continuously to the end of dendrites. The traced lengths of MAP-2+ dendrites were measured and analyzed [26, 27]. A total of ~200–300 dendrites in each area rostral or caudal to the injury site were measured and analyzed.

### Measurements of Demyelination after SCI

Measurements of SCI-induced demyelination were performed by comparing the intensity of eriochrome cyanine (EC)-stained myelin [28]. Briefly, 16 μm sections were mounted on gelatin-coated slides and dried at room temperature. Slides were placed in fresh acetone for 10 min, removed, and allowed to dry for 30 min. Sections were stained with freshly filtered EC solution (Sigma-Aldrich) for 30 min and washed in running tap water for 5 min. The stain was differentiated in 5% ferric ammonium sulphate (Sigma-Aldrich) for 15 min and washed with running tap water for 5 min. The differentiation was completed with borax-ferricyanide solution (Sigma-Aldrich) for 10 min, washed with running tap water, and allowed to dry. Slides were dehydrated in 70%, 95%, and then 100% ethanol for 2 min each, followed by xylenes for 2 min. Slides were coverslipped with a VectaMount Permanent Mounting Medium (Vector Laboratories). All images were taken using a bright field microscope (DM6000; Leica Microsystems) and analyzed using ImageJ software.

### Statistical Analysis

All values are presented as mean ± SEM. Statistical significance (p<0.05 for all analyses) between groups was assessed by one-way ANOVA using GraphPad Prism 5.01 (GraphPad, San Diego, CA, USA), followed by Student–Newman–Keuls analyses. All experimental procedures and data analyses were performed in a blinded fashion for the entire study.

## Results

### IL-6 Treatment Induced SOCS3 Expression in NS-1 Cells

**Fig 1:**
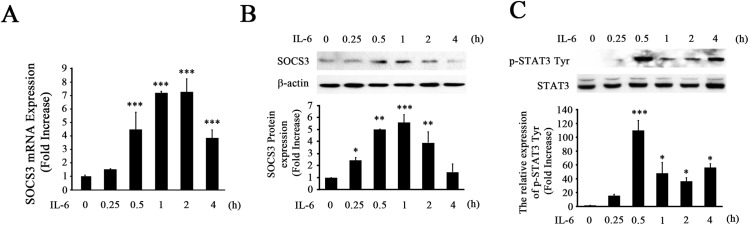
Induction of SOCS3 expression by IL-6 in NS-1 cells. NS-1 cells were treated with IL-6 for the time indicated and mRNA and protein were then analyzed by qRT-PCR and immunoblotting, respectively. A, SOCS3 mRNA expression was significantly increased after IL-6 treatment. B, Protein expression of SOCS3 was significantly increased after IL-6 treatment. C, IL-6 induced STAT3 activation by NS-1 cells. The densitometric ratios of P-STAT3 Tyr705 versus total STAT3 were calculated and are shown as fold increases. Graphs represent the mean ± SEM of triplicate culture dishes per group at each time point from four separate experiments. *p<0.05, **p < 0.01, and ***p < 0.001 compared to control, untreated cultures (one-way ANOVA and Student–Newman–Keuls analyses).

Gp130 is the common signal transducing receptor for all IL-6 family members [29, 30]. Binding of IL-6 to gp130 receptor induces SOCS3 expression as a transcription target gene of the STAT3 signaling pathway [29]. Thus, we tested whether SOCS3 expression was regulated upon IL-6 treatment in NS-1 cells. Without IL-6 treatment, basal expression levels of SOCS3 mRNA were very low. However, after IL-6 treatment, SOCS3 mRNA expression was significantly increased at 30 min, peaked at 1–2 h, and remained at a high level until 4 h (Fig 1A). SOCS3 protein expression was also significantly increased between 0.25–2 h in NS-1 cells upon IL-6 treatment (Fig 1B).

A number of studies have demonstrated that SOCS3 expression can be induced by STAT3 activation [14, 21, 31], indicating that SOCS3 is a STAT3-inducible gene. Thus, we investigated whether IL-6 induces phosphorylation of STAT3, the indicator of STAT3 activation, in NS-1 cells as evidence of an essential signaling pathway for SOCS3 expression. Immunoblotting analyses demonstrated that IL-6 induced significant increases in P-STAT3 Tyr705 within 30 min, which was maintained up to 4h (Fig 1C), while total STAT3 expression was not significantly altered following treatment.

### Reduction of SOCS3 Expression Enhanced Neurite Outgrowth in NS-1 Cells

**Fig 2:**
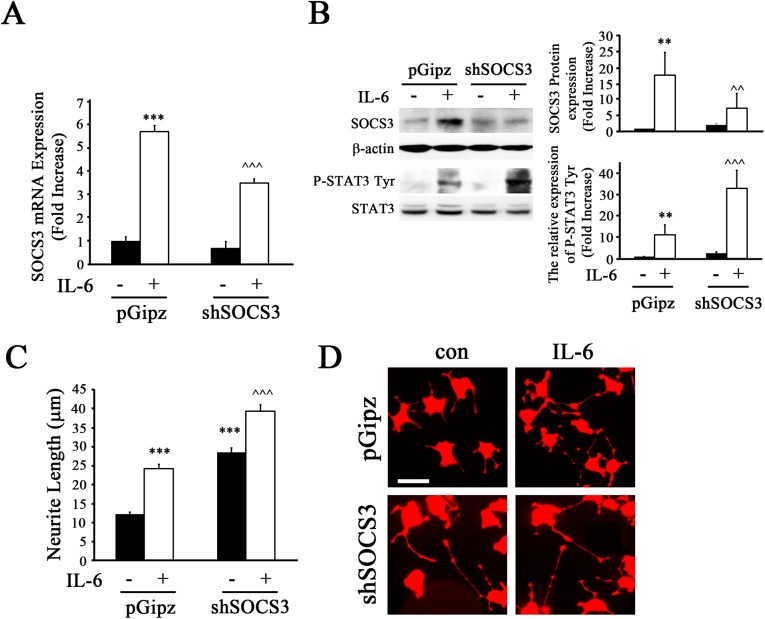
Reduced SOCS3 expression contributed to neuritic outgrowth in NS-1 cells. NS-1 cells were infected with Lenti-shSOCS3 (shSOCS3) or Lenti-pGipz (pGipz), then treated with IL-6 for 1 h, and harvested for analyses by qRT-PCR of SOCS3 mRNA expression (A) or by immunoblotting of SOCS3, P-STAT3 Tyr705, or total STAT3 (B). The densitometric ratios of P-STAT3 Tyr705 versus total STAT3 were calculated and are shown as fold increases. Graphs represent the mean ± SEM of triplicate cultures per group at each time point from three separate experiments. **p<0.01, ***p<0.001 compared to control and Lenti-pGipz-infected cultures; ^^p<0.01, ^^^p<0.001 compared to IL-6-treated and Lenti-pGipz-infected cultures. C, NS-1 cells were infected with Lenti-shSOCS3 or Lenti-pGipz and then treated with IL-6 for 3 days. The length of neurites per cell was quantified using LAS AF software. Graphs represent the mean ± SEM of triplicate culture dishes per group at each time point from three separate experiments. ***p<0.001 compared to control and Lenti-pGipz-infected; ^^^p<0.001 compared to IL-6-treated and Lenti-pGipz-infected cultures. D, Representative images of NS-1 cells from Lenti-shSOCS3- or Lenti-pGipz-infected cultures, with or without IL-6 treatment. Scale bar, 25 μm.

Recent studies demonstrate that genetic knockout of SOCS3 expression can promote axon regeneration after optic nerve injury [32], indicating that SOCS3 is as an intrinsic barrier and has a negative regulatory effect on axonal regeneration. In addition, it has been shown in both *in vitro* and *in vivo* studies that IL-6 treatment can enhance nerve growth through the JAK/STAT3 pathway [33, 34]. Thus, we investigated the regulation of SOCS3 on IL-6-induced neurite outgrowth by NS-1 cells. First, we investigated whether IL-6-induced SOCS3 expression is inhibited by lentivirus-delivered shRNA targeting endogenous SOCS3 (shSOCS3). To test this, NS-1 cells were infected with Lenti-shSOCS3 or Lenti-pGipz and then treated for 1 h with IL-6. qPCR analysis showed that shSOCS3 expression by Lenti-shSOCS3 infection led to a significant reduction in IL-6-induced SOCS3 mRNA expression (Fig 2A). Comparable to these results, SOCS3 protein expression was also decreased by Lenti-shSOCS3 upon IL-6 treatment (Fig 2B) as compared to those cells infected with Lenti-pGipz. However, Lenti-shSOCS3 enhanced IL-6-induced P-STAT3 Tyr705 by NS-1 cells (Fig 2B) when compared to cells exposed to Lenti-pGipz. Next, NS-1 cells were infected with Lenti-shSOCS3 or Lenti-pGipz and then treated with IL-6 to determine if SOCS3 regulates neurite outgrowth. We observed that reduced SOCS3 expression by Lenti-shSOCS3 enhanced IL-6-induced outgrowth of neurites, as compared to Lenti-pGipz-infected cells (~1.8 fold increase). However, the untreated cultures infected with Lenti-shSOCS3 also showed enhancement of neurite outgrowth compared to untreated cultures with Lenti-pGipz infection (~2 fold increase; Fig 2C and 2D). These results were also observed in uninfected cells treated with IL-6 (S1 Fig).

### Decreased SOCS3 Expression Enhanced P-STAT3 Tyr705 after Complete SCI

**Fig 3:**
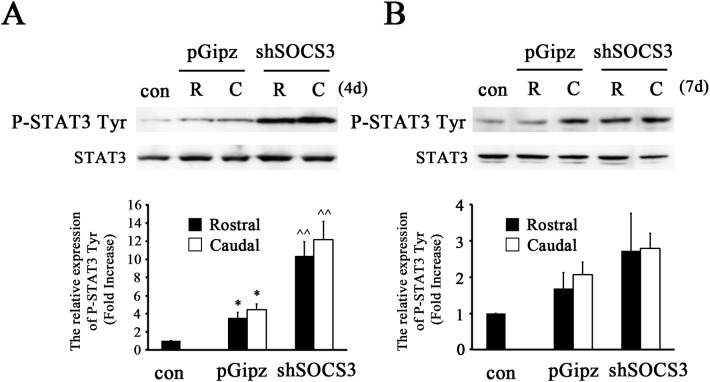
Enhancement of STAT3 activation by Lenti-shSOCS3 after complete SCI. Animals were infected with Lenti-shSOCS3 (shSOCS3) or Lenti-pGipz (pGipz) for 2 weeks and spinal cords were completely transected at the T8 level. Immunoblot analyses showed that expression of P-STAT3 Tyr705 (P-STAT3 Tyr) was enhanced by Lenti-shSOCS3 at 4 days (n = 3) (A) or 7 days (n = 3) (B) after complete SCI in areas both rostral (R) and caudal (C) to the injured site, as compared to Lenti-pGipz-infected animals (n = 3 at 4 days and n = 3 at 7 days). *p<0.05 compared to sham group; ^^p<0.01 compared to Lenti-pGipz-infected group; (one-way ANOVA and Student-Newman-Keuls analyses). N = 3 per group at each time point.

Our previous studies reported that Lenti-shSOCS3 infection in spinal cord reduced complete SCI-induced mRNA and protein expression of SOCS3, as compared to Lenti-pGipz infection [20]. We therefore tested whether SOCS3 negatively regulates SCI-induced STAT3 activation in spinal cord. As previously reported [20], we applied Lenti-shSOCS3 two weeks prior to complete SCI to decrease SOCS3 expression. The animals that received Lenti-shSOCS3 showed a significant enhancement of P-STAT3 Tyr705 at 4 days post-complete SCI, as compared to Lenti-pGipz infection; this was found similarly in both rostral and caudal spinal cord segments (Fig 3A). The enhancement of P-STAT3 Tyr705 by Lenti-shSOCS3 was not significant at 7 days after complete SCI when compared to Lenti-pGipz-infected groups (Fig 3B).

### Reduction of SOCS3 Expression Increased MAP-2+ Dendrites after Complete SCI

**Fig 4:**
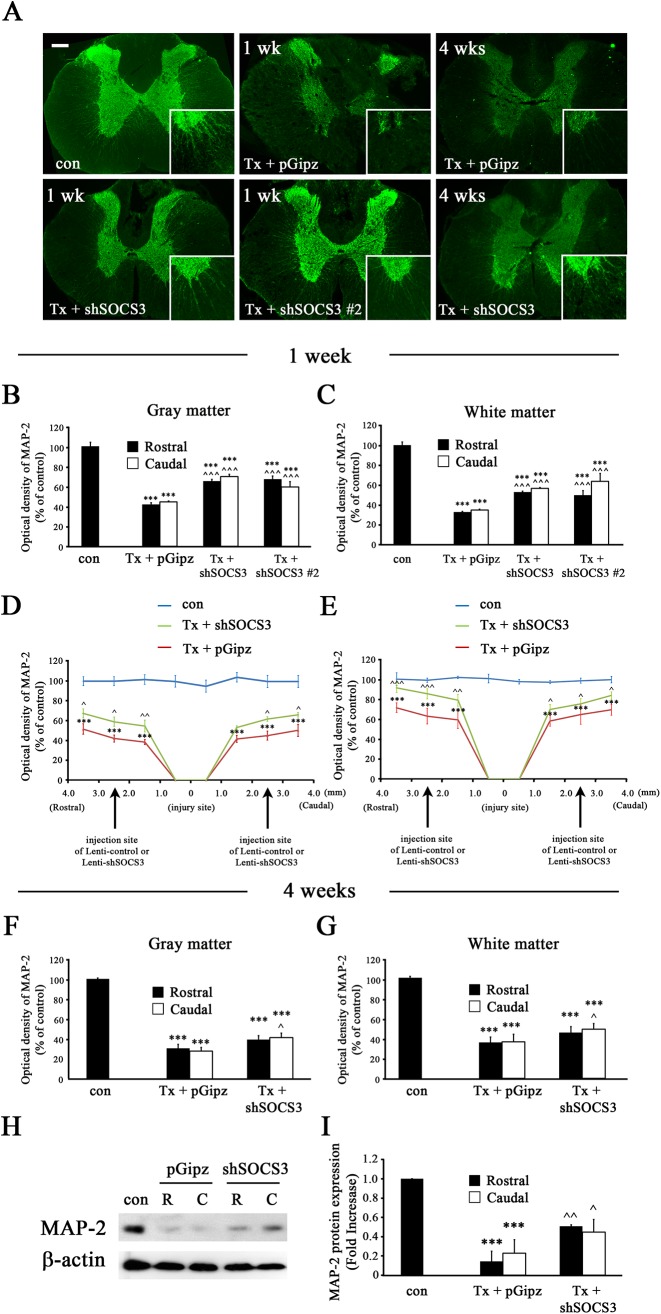
Distribution of MAP-2+ dendrites in the spinal cord after T8 complete SCI. A, Representative images of a spinal cord transverse section (rostral to the injured site) from sham (con; n = 3), Lenti-shSOCS3- (Tx + shSOCS3, n = 6), Lenti-shSOCS3 #2- (Tx + shSOCS3 #2, n = 3) or Lenti-pGipz-infected (Tx + pGipz, n = 4) animals showing that both Lenti-shSOCS3 and Lenti-shSOCS3 #2 significantly decreased SCI (Tx)-induced loss of MAP-2+ dendrites 1 week after SCI, but such effects were attenuated by Lenti-shSOCS3 at 4 weeks (n = 3 for con, n = 4 for Tx + pGipz, n = 4 for Tx + shSOCS3) post-injury. Scale bar, 250 μm. Quantification of MAP-2 immunoreactive intensity in spinal cords harvested at 1 (B-E) or 4 weeks (F-G) after SCI with Lenti-shSOCS3, Lenti-shSOCS3 #2, or Lenti-pGipz infection were analyzed in either gray (B, D, and F) or white matter (C, E, and G). The immunoreactivities of MAP-2 in both gray (B, D, and F) and white matter (C, E, and G) following Lenti-shSOCS3 infection were significantly higher than those following Lenti-pGipz infection in areas both rostral and caudal to the injured site both 1 week (B-E) and 4 weeks (F-G) after SCI. ***p < 0.001 compared to sham control; ^p<0.05, ^^p<0.01, and ^^^p < 0.001 compared to Lenti-pGipz infection (one-way ANOVA and Student-Newman-Keuls analyses). H-I, Immunoblot analyses showed that expression of MAP-2 was enhanced by Lenti-shSOCS3 (n = 3) 1 week after complete SCI in areas both rostral (R) and caudal (C) to the injured site. ***p<0.001 compared to sham control group (n = 3); ^p<0.05 and ^^p<0.01 compared to Lenti-pGipz-infected group (n = 3); (one-way ANOVA and Student-Newman-Keuls analyses).

We next investigated if the negative regulatory effects of SOCS3 on neurite outgrowth in NS-1 cells can be also observed in spinal cord neurons after complete SCI. MAP-2 immunoreactivities after complete SCI were examined as markers of dendrite density in spinal cord [35, 36]. The spinal cord was completely transected 2 weeks after Lenti-shSOCS3 or Lenti-pGipz infection, and then 1 or 4 weeks after SCI the animal was sacrificed and immunostained with MAP-2 antibody. In sham-operated rat spinal cord (control), MAP-2 immunoreactivity was localized predominantly in dendrites within the gray matter and extended to the white matter of the spinal cord (Fig 4A). Lenti-pGipz infection followed by complete SCI significantly reduced MAP-2+ dendrites in gray matter and white matter at both 1 and 4 weeks after injury compared to control animals. However, significantly more MAP-2+ dendrites were seen in spinal cords of Lenti-shSOCS3-infected animals as compared to those with Lenti-pGipz infection (Fig 4A). Similar enhancement effects on MAP-2+ dendrites were also observed when Lentivirus delivering the second shRNA (Lenti-shSOCS3 #2), specifically knocking down SOCS3 (S2 Fig), was applied (Fig 4A). Quantitative analyses demonstrated that both Lenti-shSOCS3 and Lenti-shSOCS3 #2 infection decreased SCI-induced loss of MAP-2+ dendrites in both gray (Fig 4B) and white (Fig 4C) matter at 1 week after complete SCI when compared to Lenti-pGipz infection. These enhanced effects of Lenti-shSOCS3 on MAP-2+ dendritic immunoreactivity were significant in the areas 1.5 mm away from the injury site both in gray (Fig 4D) and white (Fig 4E) matter of spinal cord. These results strongly suggest that the effects of Lenti-shSOCS3 in protecting MAP-2+ dendrites from SCI-induced decreases are specifically mediated by reduced SOCS3 expression, rather than off target effects of shRNA [24]. However, such enhancement effects of Lenti-shSOCS3 were less prominent 4 weeks after SCI, but were still significantly observed in the caudal areas of spinal cord in both gray (Fig 4F) and white (Fig 4G) matter. Consistent with these immunostaining results, immunoblot analysis demonstrated that complete SCI with Lenti-pGipz treatment lead to significant loss of MAP-2 expression both rostral and caudal to the lesion epicenter at 1 week post-injury (Fig 4H and 4I) when compared to sham animals (con). However, complete SCI-induced loss of MAP-2 expression was inhibited by Lenti-shSOCS3 treatment in the spinal cord both rostral and caudal to the lesion epicenter (Fig 4H and 4I).

**Fig 5:**
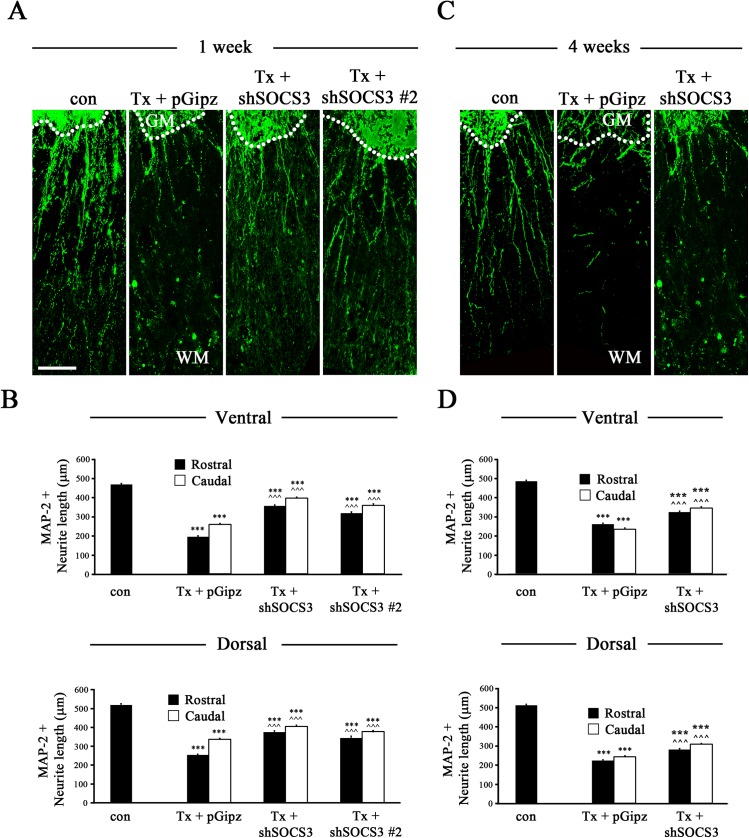
MAP-2+ dendritic length in the white matter of spinal cord after T8 complete SCI. (A) Representative images showing MAP-2+ dendrites in the ventral horn of a spinal cord transverse section (rostral to the injured site) from sham (con; n = 3), Lenti-pGipz- (Tx + pGipz, n = 4), Lenti-shSOCS3 (Tx + shSOCS3, n = 6), or Lenti-shSOCS3 #2 (Tx + shSOCS3 #2, n = 3)-infected animals at 1 week after SCI (Tx). (C) Representative images showing MAP-2+ dendrites in the ventral horn of a spinal cord transverse section (rostral to the injured site) from sham (n = 3), Lenti-pGipz- (n = 4), or Lenti-shSOCS3 (n = 4)-infected animals at 4 weeks after SCI. GM and WM indicate gray matter and white matter, respectively. Scale bar, 75 μm. Statistical analyses indicated that MAP-2+ dendritic length in both ventral horn (Ventral, the upper graphs of B and D) and dorsal horn (Dorsal, the lower graphs of B and D) following Lenti-shSOCS3 infection was significantly longer than that following Lenti-pGipz infection in areas both rostral and caudal to the injured site both 1 (B) and 4 weeks (D) after SCI. ***p < 0.001 compared to sham control; ^^^p < 0.001 compared to Lenti-pGipz infection (one-way ANOVA and Student-Newman-Keuls analyses).

To further investigate whether SOCS3 expression negatively regulates dendrite outgrowth of spinal cord neurons into the white matter after complete SCI, MAP-2+ dendritic length was analyzed. In sham control rat spinal cord (Fig 5A) MAP-2+ dendrites were long with an intact shape. However, Lenti-pGipz treatment followed by complete SCI had scattered and fragmented dendrites at both 1 (Fig 5A) and 4 (Fig 5C) weeks after injury when compared to sham control animals. In contrast, when compared to Lenti-pGipz infection, reduction of SOCS3 expression by Lenti-shSOCS3 resulted in longer MAP-2+ dendrites at both 1 (Fig 5A) and 4 (Fig 5C) weeks after SCI. As expected, similar effects were seen in the Lenti-shSOCS3 #2-infected group (Fig 5A). Quantitative analyses demonstrated that both Lenti-shSOCS3 and Lenti-shSOCS3 #2 infection increased the number of long MAP-2+ dendrites in both ventral and dorsal horn of spinal cord 1 week after complete SCI as compared to Lenti-pGipz infection (Fig 5B). Such increases/protection effects on MAP-2+ dendrites were still observed in both the ventral and dorsal horns of spinal cord 4 weeks after complete SCI as compared to Lenti-pGipz infection (Fig 5D). These data suggested that reduction of SOCS3 in spinal cord promotes local dendritic growth after complete SCI and/or prevents dendrites from SCI-induced dying back/degeneration in the white matter.

### Reduction of SOCS3 Expression Enhanced SCI-Induced GAP-43 Expression

**Fig 6:**
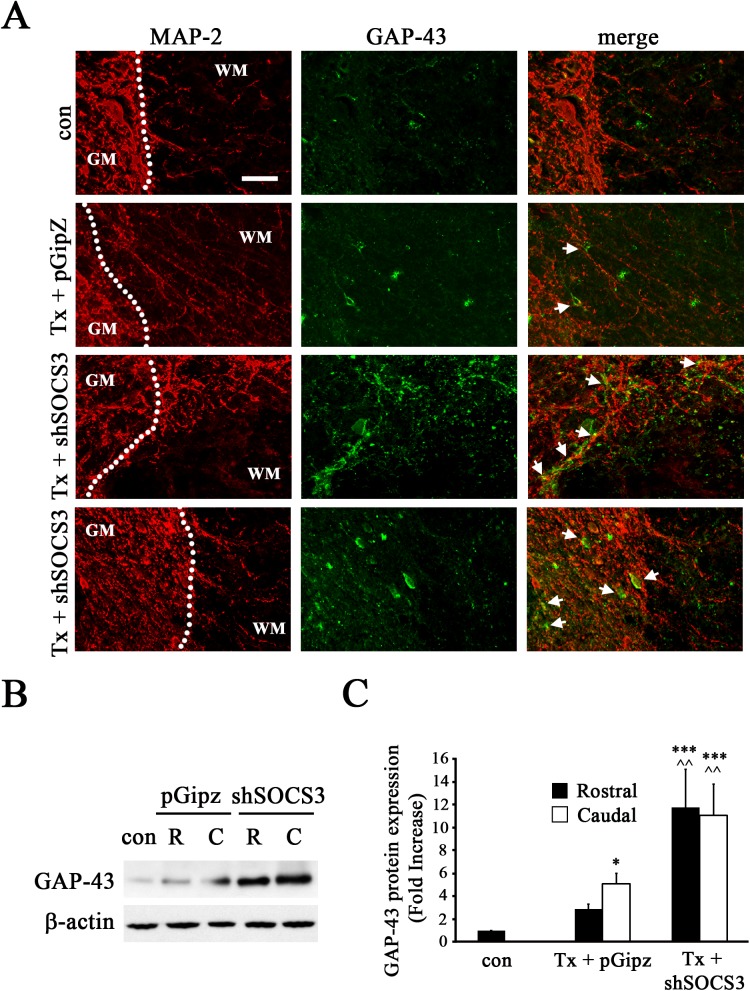
Reduced SOCS3 expression enhanced GAP-43 expression in the spinal cord after complete SCI. A, Representative images of double labeling with GAP-43 (green) and MAP-2 (red) showed that Lenti-shSOCS3 significantly enhanced GAP-43 immunoreactivities at 1 week after SCI (Tx + shSOCS3, n = 6). Note the increased co-localization of MAP-2+ dendrites or cell bodies with GAP-43 in the Lenti-shSOCS3-infected group (arrow) as compared to the Lenti-pGipz group (Tx + pGipz, n = 4). N = 6 for control (con) group. Scale bar, 25 μm. B-C, Immunoblot analyses showed that expression of GAP-43 was enhanced by Lenti-shSOCS3 (shSOCS3, n = 3) at 1 week after complete SCI in areas both rostral (R) and caudal (C) to the injured site. *p<0.05, and ***p<0.001 compared to sham group; ^^p<0.01 compared to Lenti-pGipz-infected group (pGipz, n = 3) (one-way ANOVA and Student-Newman-Keuls analyses).

To clarify whether Lenti-shSOCS3 infection decreasing SCI-induced loss in MAP-2+ dendrites is attributable to increased growth/regeneration or decreased dying back/degeneration on local dendrites, MAP-2+ dendrites were further double-stained with GAP-43, a marker for neural regeneration. As expected, basal levels of GAP-43 in spinal cord were very low in sham control animals (Fig 6A). GAP-43 expression was slightly induced after complete SCI in Lenti-pGipz-infected spinal cord, which was co-localized with MAP-2+ dendrites (Fig 6A). Significant increases in the expression of GAP-43 were induced in the spinal cord of Lenti-shSOCS3-infected animals (Fig 6A), as compared to those with Lenti-pGipz infection. Moreover, the increased GAP-43 was co-localized with MAP-2+ dendrites in white matter (third row, Fig 6A) and/or co-localized with MAP-2+ cell bodies in gray matter (fourth row, Fig 6A) in Lenti-shSOCS3-infected spinal cord. Consistent with these results, immunoblot analysis demonstrated that complete SCI with Lenti-pGipz treatment induce a slight increase in GAP-43 expression both rostral (2.8-fold increase) and caudal (5-fold increase) to the lesion epicenter at 1 week post-injury (Fig 6B and 6C) when compared to sham control animals. Notably, SCI-induced GAP-43 expression was even more significant and dramatic with Lenti-shSOCS3 infection, as compared to the increases induced by Lenti-pGipz infection in the spinal cord both rostral (4-fold increase) and caudal (2.2-fold increase) to the lesion epicenter (Fig 6B and 6C).

### Prevention of Demyelination by Reducing SOCS3 Expression after Complete SCI

**Fig 7:**
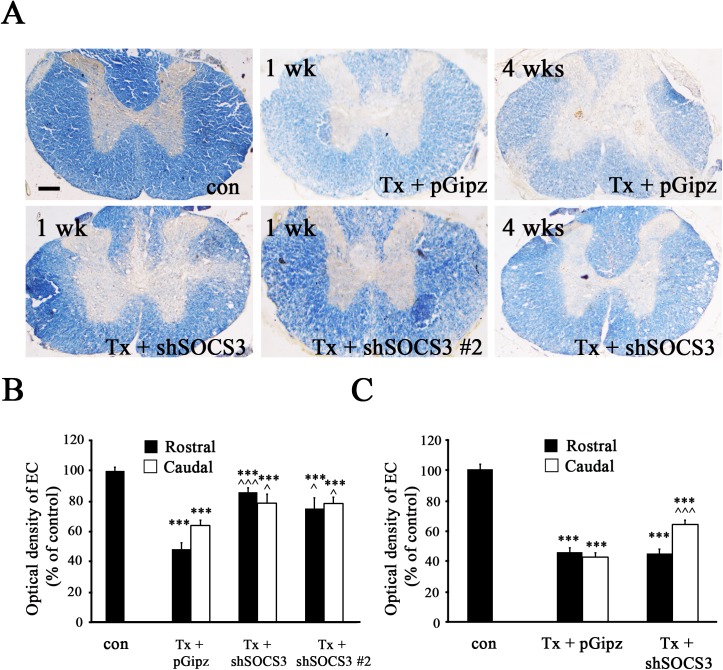
Reduced SOCS3 expression decreased SCI-induced demyelination in white matter of spinal cord. A, Representative images showed EC-stained myelin in transverse spinal cord sections (rostral to the injured site) from sham (con), Lenti-pGipz- (Tx + pGipz), Lenti-shSOCS3-infected (Tx + shSOCS3), or Lenti-shSOCS3 #2 (Tx + shSOCS3 #2) animals at 1 week (n = 3 for con, n = 4 for Tx + pGipz, n = 6 for Tx + shSOCS3, n = 3 for Tx + shSOCS3 #2) or 4 weeks (n = 3 for con, n = 4 for Tx + pGipz, n = 4 for Tx + shSOCS3) after SCI (Tx). Scale bar, 250 μm. Statistical analyses indicated that the intensity of EC-stained myelin following Lenti-shSOCS3 or Lenti-shSOCS3 #2 infection was significantly increased compared to Lenti-pGipz infection 1 week after SCI in areas both rostral and caudal to the injured site (B) and was only significantly increased in caudal spinal cord when harvested 4 weeks after SCI (C). ***p < 0.001 compared to sham control; ^p<0.05, and ^^^p < 0.001 compared to the Lenti-pGipz-infected group (one-way ANOVA and Student-Newman-Keuls analyses).

The STAT3/SOCS3 pathway has been shown to be involved in neuroprotection [20, 21, 23] and protection against demyelination [37]. In the present study, we investigated whether reduction of SOCS3 expression by Lenti-shSOCS3 can decrease SCI-induced demyelination. To test this, EC binding to myelin was used to stain spinal cord sections harvested 1 or 4 weeks after complete SCI with either Lenti-shSOCS3 or Lenti-pGipz infection. Spinal cords from Lenti-pGipz infection displayed significant demyelination at both 1 (Fig 7A and 7B) and 4 (Fig 7A and 7C) weeks after complete SCI. In contrast, demyelination was significantly reduced in both Lenti-shSOCS3-and Lenti-shSOCS3 #2-infected spinal cord 1 week after complete SCI in areas both rostral and caudal to the injured site (Fig 7A and 7B). Even 4 weeks after SCI, Lenti-shSOCS3 infection still caused a significant reduction in demyelination in areas caudal to the injured site compared to Lenti-pGipz infection (Fig 7A and 7C). These results support the concept that Lenti-shSOCS3 decreased demyelination after complete SCI specifically by reducing SOCS3 expression.

## Discussion

In the present study, we first investigated the expression of SOCS3 by NS-1 cells in response to IL-6, the regulatory effect of SOCS3 on STAT3 activation, and the role of the SOCS3/STAT3 pathway in neuritic outgrowth. IL-6 treatment induced SOCS3 expression in NS-1 cells and led to STAT3 activation. SOCS3 negatively regulates STAT3 activation, which in turn inhibits IL-6-induced neuritic outgrowth *in vitro*. Lenti-shSOCS3 could block such negative regulation by reducing SOCS3 expression. We further investigated the role of knocked down SOCS3 in promoting dendritic growth/regeneration and myelination after complete SCI. Reduction of SOCS3 by Lenti-shSOCS3 increased MAP-2+ dendrites (both density and length) at both 1 and 4 weeks after SCI. Lenti-shSOCS3 decreased SCI-induced demyelination in white matter at 1 and 4 weeks post-injury. The current results are the first to demonstrate that (1) endogenous SOCS3 expression by NS-1 cells contributes to inhibition of IL-6-induced neuritic growth, (2) reduction of SCI-increased SOCS3 expression by Lenti-shSOCS3 in spinal cord neurons increases GAP-43 expression and enhances MAP-2+ dendritic growth in white matter after complete SCI, and (3) reduction of SOCS3 expression prevents further demyelination after T8 complete SCI in adult rats.

Activated STAT3 functions as a key effector of neuritic outgrowth via transcriptional activation [22, 38, 39]. In dorsal root ganglion (DRG) neurons, over-expression of STAT3 increases neuritic growth beyond baseline levels [22]. In addition, inhibition of P-STAT3 Tyr705 after sciatic nerve transection resulted in reduced neuritic outgrowth *in vitro* [39]. In contrast, SOCS3 is known to limit IL-6-mediated signaling by inhibiting JAK tyrosine kinase activity, thereby preventing STAT3 activation. SOCS3 expression in neurons plays a negative role in regulating neuritic outgrowth [22, 40]. In DRG neurons, over-expression of SOCS3 inhibits neuritic outgrowth [22]. Smith et al. [32] reported that in optic nerve, conditional deletion of SOCS3 promoted regeneration of injured optic nerve axons. In SOCS3-gp130 double knockout mice, the regeneration effect was ablated, suggesting that the effects of SOCS3 deletion in regulating optic nerve regeneration are mediated by gp130 [32], the common signal transducing receptor for all IL-6 family members [29, 30]. In our study, reduced SOCS3 expression enhanced IL-6-induced neuritic outgrowth via STAT3 activation in NS-1 cells. Interestingly, the control cultures without IL-6 treatment had enhanced neurite growth when infected by Lenti-shSOCS3. These results might be caused by serine phosphorylation of STAT3 in mitochondria, which is non-transcriptional regulation, leading to neurite outgrowth. A recent report demonstrated that NGF treatment induced serine phosphorylation of STAT3, but not tyrosine phosphorylation, which regulates neurite growth in PC12 cells [41]. Consistent with these results, our unpublished data show that Lenti-shSOCS3 can regulate not only tyrosine phosphorylation of STAT3, but also serine phosphorylation.

Moreover, we observed that reduction of SOCS3 by Lenti-shSOCS3 in rat spinal cord neurons enhances MAP-2+ dendritic growth after complete SCI. These findings suggest that SOCS3 negatively regulates neuritic growth by inhibiting IL-6-induced P-STAT3 Tyr705. In addition, Lenti-shSOCS3 infection results in more and longer MAP-2+ dendrites after complete SCI. Reduction of SOCS3 by Lenti-shSOCS3 increased both (1) growth/regeneration-promoting STAT3 activities, and (2) GAP-43 expression after complete SCI. Collectively, these findings suggest that knocked down SOCS3 promotes local dendritic growth/regeneration. However, the possibility cannot be ruled out that Lenti-shSOCS3 may also prevent further dying back/degeneration to maintain dendrite density and/or length, as dendrite lengths decreased over time due to SCI-induced degeneration. These results coincide with several other reports showing that SOCS3 is an intrinsic factor for the negative regulation of axonal regeneration after injury in the central nervous system (CNS) [22, 32, 42]. Furthermore, our previous studies demonstrated that reduction of SOCS3 protects neurons from cell death after SCI [20]. Specifically, treatment with Lenti-shSOCS3 results in not only a greater number of neurons, but also more healthy neurons after complete SCI. Thus, the enhancement effects of reduced SOCS3 on MAP-2+ dendritic outgrowth in spinal cord after SCI might be partially attributable to a greater number of healthy neurons surviving after SCI.

The distributions of dendritic arborizations in the white matter of the CNS are critical for behavioral functions. For instance, reticular neurons may project dendrites into the adjacent corticospinal tract [43]. The subthalamic nucleus has dendritic projections to the cerebral peduncle, where they receive inputs from the ascending pathways [44]. In the current study, we demonstrated that there is a significant reduction of MAP-2+ dendritic projections in white matter after complete SCI and that reduction of SOCS3 expression in spinal cord neurons can decrease SCI-induced loss of MAP-2+ dendritic projections in terms of both dendritic density and length. Further studies will be needed to determine whether these dendrites make any synaptic contacts with descending or ascending pathways in the white matter of spinal cord.

The function of SOCS3 in the brain is cell type-specific [14, 31]. Several lines of evidence suggest that in microglia, SOCS3 inhibits cytokine-induced immune and inflammatory responses *in vitro* [45, 46] and that in astrocytes, SOCS3 enhances inhibition of chemokine expression and T-cell migration [31], suggesting that SOCS3 expression in macrophages, microglia, and astrocytes suppresses brain inflammation. However, the role of SOCS3 in oligodendrocytes appears to be detrimental by inhibiting STAT3 activation and subsequent downstream neuroprotective effects [47]. LIF and CNTF play a protective role in oligodendrocyte survival *in vitro* [48] and *in vivo* [49]; furthermore, in an experimental autoimmune encephalomyelitis (EAE) animal model of multiple sclerosis (MS), double deletion of LIF-R and gp130 to inhibit STAT3 activation worsened MS-induced demyelination when compared to wild-type animals [50]. Our previous study showed that infection of Lenti-shSOCS3 into rat spinal cord is mainly distributed in neurons [20]. The present study demonstrates that Lenti-shSOCS3 infection decreases complete SCI-induced demyelination in white matter of spinal cord. The neuroprotective effect of Lenti-shSOCS3 infection may lead to reduced demyelination. Collectively, targeting the SOSC3/STAT3 pathway may provide a potential therapeutic strategy following SCI.

## Supporting Information

S1 Fig IL-6 induced neurite outgrowth in NS-1 cells. A, NS-1 cells were treated with IL-6 for 3 days and then the length of neurites of NS-1 cells was quantified using LAS AF software. Graphs represent the mean ± SEM of triplicate cultures in three separate experiments. ***p<0.001 compared to untreated controls. B, Representative images of NS-1 cells from untreated (con) or IL-6-treated cultures (IL-6). Scale bar, 25 μm.
(TIF)

S2 Fig Reduction of SOCS3 mRNA expression using lentiviral plasmids encoding either shSOCS3 or shSOCS3 #2, which are two different shRNAs specific to SOCS3. SHSY-5Y cells were infected with Lenti-pGipz (pGipz), Lenti-shSOCS3 #2 (shSOCS3 #2), or Lenti-shSOCS3 (shSOCS3), and then treated with Oncostatin M (OSM) for 1 h. mRNA was then analyzed by qPCR for SOCS3 mRNA expression. Graphs represent the mean ± SEM of triplicate cultures in three separate experiments. ***p<0.001 compared to untreated controls; ^^^p<0.001 compared to OSM-treated Lenti-pGipz-infected cultures.
(TIF)
