# Supplementary figures and images for: Effects of Reducing Suppressors of Cytokine Signaling-3 (SOCS3) Expression on Dendritic Outgrowth and Demyelination after Spinal Cord Injury
Source: PLoS One. 2015 Sep 18;10(9):e0138301. doi: 10.1371/journal.pone.0138301 (PMC4575181; doi:10.1371/journal.pone.0138301)

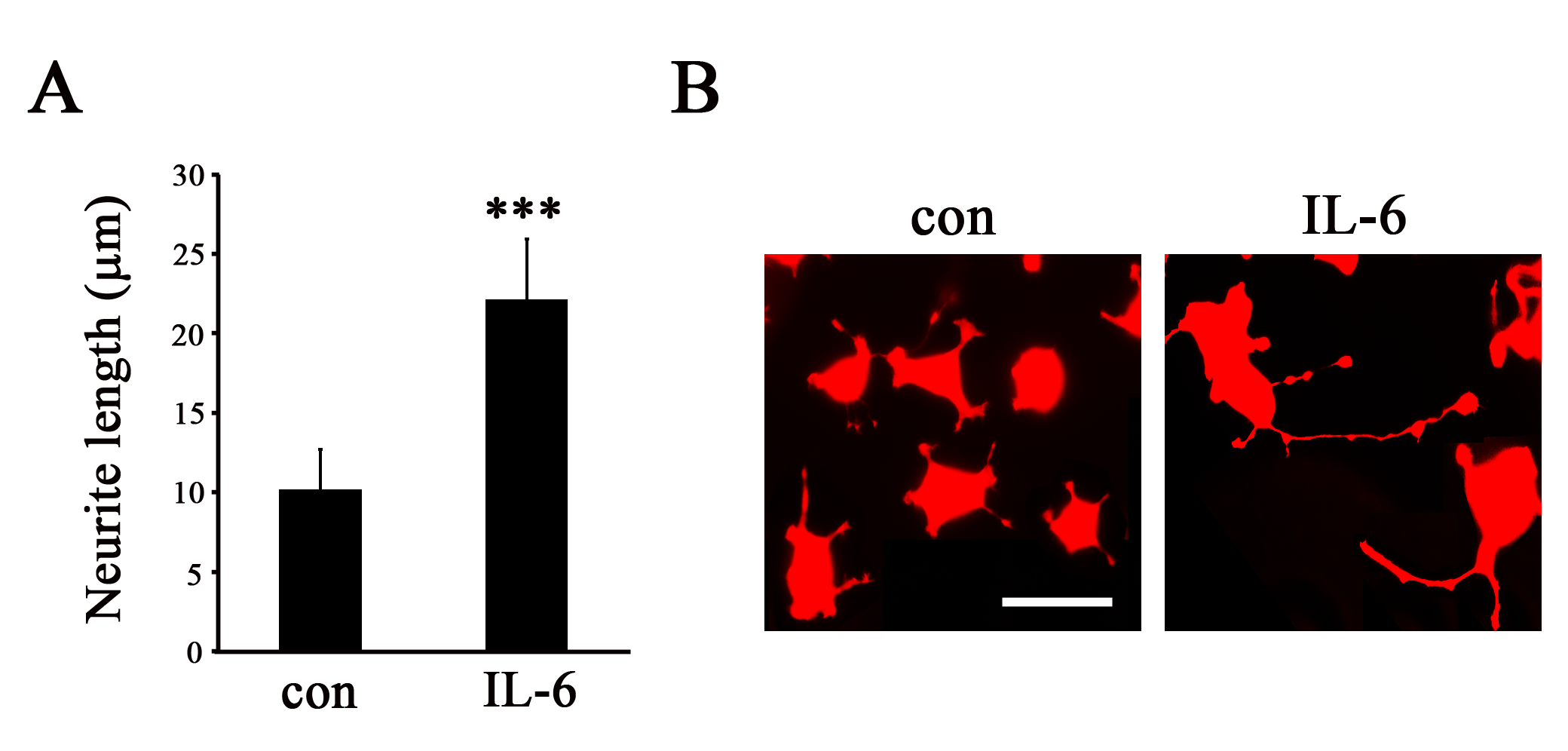

Supplement: S1 Fig — A, NS-1 cells were treated with IL-6 for 3 days and then the length of neurites of NS-1 cells was quantified using LAS AF software. Graphs represent the mean ± SEM of triplicate cultures in three separate experiments. ***p<0.001 compared to untreated controls. B, Representative images of NS-1 cells from untreated (con) or IL-6-treated cultures (IL-6). Scale bar, 25 μm. (TIF) [file pone.0138301.s001.tif]

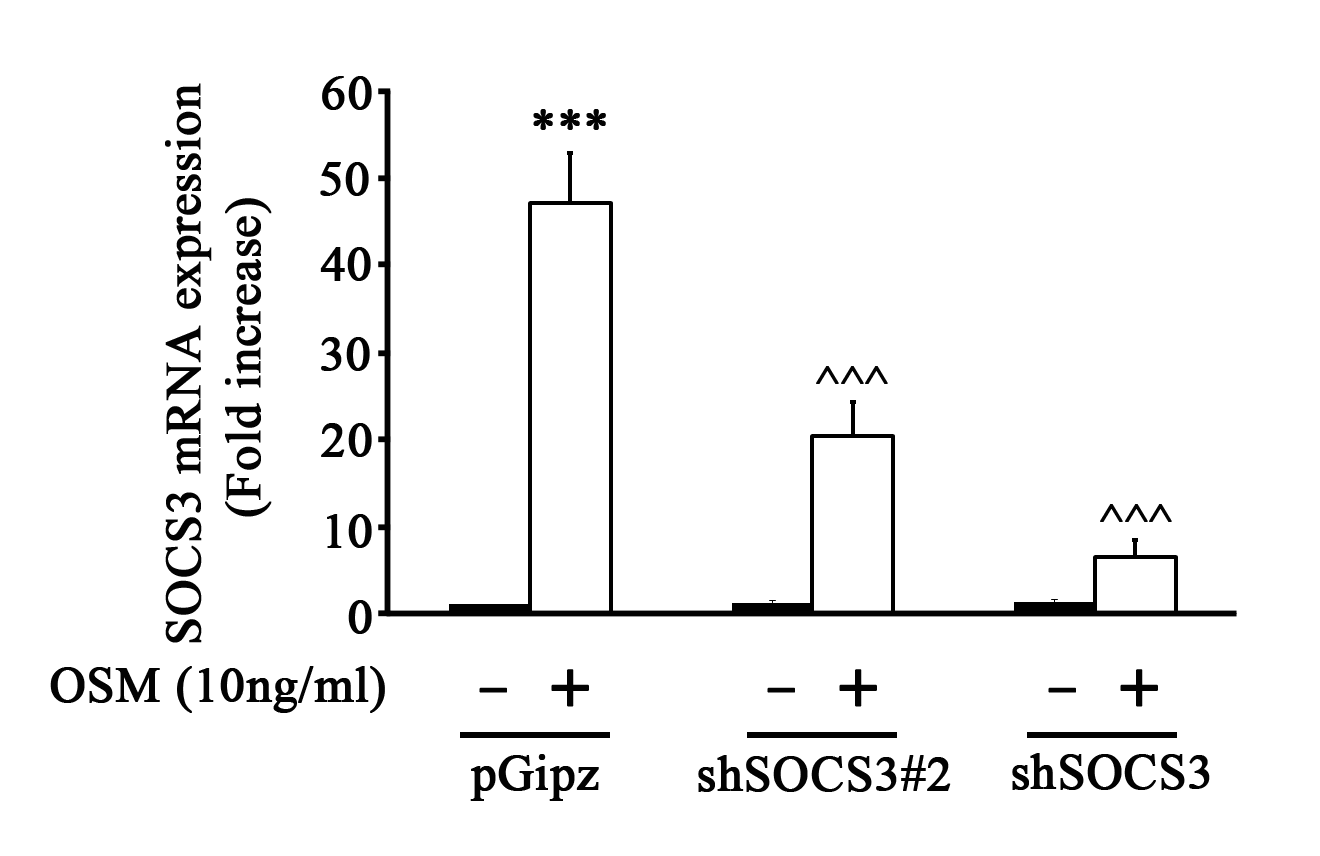

Supplement: S2 Fig — SHSY-5Y cells were infected with Lenti-pGipz (pGipz), Lenti-shSOCS3 #2 (shSOCS3 #2), or Lenti-shSOCS3 (shSOCS3), and then treated with Oncostatin M (OSM) for 1 h. mRNA was then analyzed by qPCR for SOCS3 mRNA expression. Graphs represent the mean ± SEM of triplicate cultures in three separate experiments. ***p<0.001 compared to untreated controls; ^^^p<0.001 compared to OSM-treated Lenti-pGipz-infected cultures. (TIF) [file pone.0138301.s002.tif]
